# Topical Collection: New Insights on Sphingolipids in Health and Disease

**DOI:** 10.3390/ijms24119528

**Published:** 2023-05-31

**Authors:** Andrea Huwiler

**Affiliations:** Institute of Pharmacology, University of Bern, Inselspital, INO-F, CH-3010 Bern, Switzerland; huwiler@pki.unibe.ch; Tel.: +41-31-632-32-14

The last two decades have boosted research on sphingolipids as bioactive and signaling molecules. Certain sphingolipid species, such as ceramide, sphingosine, and sphingosine 1-phosphate (S1P), are now well appreciated as key regulators of fundamental physiological and pathophysiological processes including cell proliferation and survival, cell death, cell differentiation, migration, tissue remodeling and immune cell trafficking [1,2,3]. Therefore, the enzymes that build up and degrade these sphingolipids, or convert them into each other, have attracted a lot of interest in the pharmaceutical community as they could represent novel drug targets for the treatment of diseases associated with abnormal cell growth and death, disturbed immunity and organ fibrosis.

This topical collection includes a series of original research and review articles that bring new insights on the important role of S1P in different physiological and pathophysiological settings, such as the BBB barrier regulation and immune cell homing, two important mechanisms of neuroinflammatory diseases; the role of hypothalamic S1P in feeding and fasting behavior; the role of S1P in inflammatory skin diseases and in renal erythropoietin synthesis; the role of ceramide kinase and ceramide 1-phosphate (C1P) in metastatic mechanisms of cancer cells; the contribution of sphingolipids to the cytotoxic effect of the c-Raf inhibitor sorafenib on colon cancer cells; and the role of neutral sphingomyelinase in wound healing mechanisms. Furthermore, sphingolipids also serve as lead structures for novel synthetic compounds that could be used therapeutically to treat various diseases such as cancer, inflammation, autoimmune diseases, and organ fibrosis.

In the study by Stepanovska et al. [4], two novel morpholino derivatives of fingolimod, named ST-1983 and ST-1984, were synthesized and characterized as selective and full agonists at the S1P_1_ receptor with subsequent functional antagonistic activities in the cellular system and in vivo. In the experimental antigen-induced encephalomyelitis (EAE) model in mice, both compounds induced lymphopenia and reduced disease symptoms. It was concluded that due to their S1P_1_-selective nature, these novel morpholino compounds would exhibit fewer adverse effects than fingolimod and thus may have an advantage over fingolimod in the treatment of multiple sclerosis patients, but also for other autoimmune diseases.

S1P_1_ receptor activation has a clear therapeutic effect in relapsing-remitting multiple sclerosis by involving different mechanisms including the trapping of cytotoxic T cells in secondary lymphoid organs, increasing the BBB integrity, and mediating direct neuroprotection [5,6]. In another study by Stepanovska et al. [7], the in vitro effect of intracellular S1P on the BBB build-up and breakdown was addressed by using a human cerebral microcapillary endothelial cell line. Accumulation of intracellular S1P was obtained by generating a stable knockdown cell line of the S1P lyase (SPL-kd). Results showed that in unstimulated SPL-kd cells, the transendothelial electrical resistance, as a measure of barrier integrity, built up more slowly and was destabilized more quickly. However, in an inflammatory setting, the loss of SPL mediated a protection from proinflammatory cytokine-mediated barrier breakdown. It was concluded that SPL modulation could be a valid approach to dampen an inflammatory response and enhance barrier integrity during an inflammatory challenge in brain endothelial cells.

Stroke is another disease with a strong neuroinflammatory component that is characterized by a disrupted BBB function [8,9]. Thus, in mouse models of focal cerebral ischemia, an acute inflammatory response is triggered by infiltrated inflammatory cells and T cells into the ischemic brain that contributes to post-ischemic brain damage. In the study by Lucaciu et al. [10], the concentrations of S1P and other sphingolipids were quantified in secondary lymphoid organs, in circulation and in the brain after middle cerebral artery occlusion (MCAO) in mice, allowing for new information on the kinetics of the S1P metabolism and the differential regulation of S1P receptors on systemic alterations of immune cell populations with reference to their cerebral recruitment in the acute phase of ischemic stroke. Results showed a steep S1P gradient towards the brain, with the lowest concentration being in the spleen, moderate concentration in the circulation, and highest concentration in the ischemic core of the brain, 24 h after MCAO in mice. The S1P gradient correlated with splenic S1P_1_ expression and T cell egress. The evading T cells were characterized as T_H_ and T_REG_ cells. It was concluded that after acute ischemic stroke, S1P receptors are organ-specifically regulated to allow lymphocyte subpopulations recruitment towards the peri-infarct region of the brain and systemic stroke-induced immunosuppression.

The hypothalamus is the main center for controlling feeding and fasting behavior and energy metabolism [11]. This is accomplished by secreting various orexigenic or anorexic neuropeptides, which can interact functionally to regulate feeding and thus maintain energy homeostasis [12]. While the role of such neuropeptides has been well studied, the role of lipids, and especially of sphingolipids, in hypothalamic regulation and energy homeostasis is little understood. The study by Vorzella et al. [13] investigated the effect of feeding, fasting, and refeeding on changes of S1P levels in the hypothalamus of mice. Results suggested that feeding increases hypothalamic S1P levels, while food deprivation suppresses S1P levels through a mechanism involving a transcriptional effect on enzymes in the sphingolipid biosynthetic pathway. These findings support the idea of S1P acting as a central satiety factor and that an elevation of S1P is regulated by the feeding status.

A novel physiological role of S1P in kidney function was presented by Hafizi et al. [14]. By using an immortalized mouse cell line of renal Epo-producing cells (REPC) that was recently isolated [15], they showed that S1P and the approved S1P receptor modulator fingolimod both stimulated Epo synthesis via a preceding stabilization of HIF-2α. Since it is well known that patients suffering from chronic kidney disease (CKD) will develop anemia due to reduced synthesis of erythropoietin (Epo) in the kidney, the finding by Hafizi et al. may open a new pharmacological strategy to increase renal Epo synthesis and trigger erythropoiesis in CKD patients suffering from anemia.

Sphingolipids also play important structural roles in the skin [16]. In particular, very long-chain ceramides are crucial for the skin barrier function. Recently, it has emerged that S1P is also involved in processes such as the proliferation and differentiation of keratinocytes, and it exerts an immunomodulatory role in skin [17]. The review by Kleuser and Bäumer [18] comprehensively summarizes the role of S1P in inflammatory and pruritic skin conditions and highlights the potential of targeting the S1P/S1PR signaling axis for innovative therapeutic approaches in this field.

Notably, vitamin D_3_ is used to treat hyperproliferative skin diseases. It acts by inducing the growth arrest and differentiation of keratinocytes while preventing apoptosis of keratinocytes. These events were suggested to involve Sphk activation and S1P formation [19].

In another setting such as skin wound healing, keratinocyte proliferation is needed. In the study by Patria et al. [20], it was shown that vitamin D_3_ improved wound healing and protected against UVB-induced damage in human keratinocytes, and this effect critically involved neutral sphingomyelinase (nSMase) protein and mRNA upregulation and activation.

Sphingolipids have also been associated with tumor pathologies, and while S1P can mediate tumor growth and progression, ceramide is rather linked to chemosensitization and tumor cell death [21,22]. More recently, a pro-tumorigenic effect has also been attributed to another phosphorylated sphingolipid species, C1P, which is generated intracellularly by the action of a ceramide kinase (CerK) [23]. However, the detailed mechanisms and targets of intracellular C1P promoting cancer cell growth and progression remain little understood.

In the study by Schwalm et al. [24], the human breast cancer cell line MDA-MB-231, and two thereof derived highly metastatic sublines from lung and bone, were used to characterize the contribution of CerK to the metastatic potential of cells. The authors demonstrated that CerK substantially contributed to breast cancer migration and invasion through activation of the PI3K/Akt and the Rho kinase pathways. In addition, a highly potent CerK inhibitor, NVP-231, reduced cell migration and invasion.

In another study by Jakobi et al. [25], the effect of the c-Raf inhibitor sorafenib on sphingolipid levels in hepatocellular carinoma cells (HCC) was investigated. Sorafenib is to date the most frequently applied systemic drug for HCC therapy [26], and it was hypothesized that this drug may act by altering the sphingolipid content of HCC and thereby contributing to the cytotoxic effect of the drug. Results showed that sorafenib had antiproliferative effects and enhanced dihydroceramides levels in HCC in vitro, but the inhibition of various enzymes of the sphingolipid metabolism neither abrogated nor potentiated the effects of sorafenib, thus excluding that sphingolipids are involved in the sorafenib-mediated cytotoxic effect.

Finally, in a very timely review article, Abu-Farha et al. [27] comprehensively reviewed the state-of-the-art knowledge on the role of lipids in COVID-19 virus infection and the possible involvement of lipids in the fusion of the viral membrane to the host cell, in viral replication, and in viral endocytosis and exocytosis. They also highlighted the possibility to use sphingolipid-related therapeutics such as S1P receptor modulators for the treatment of COVID-19 infections. While clinical trials were initiated rapidly after the pandemic outbreak, only a few of these planned trials were finalized.

Among these is a single-center, non-randomized controlled clinical study, which included 40 patients with moderate to severe COVID-19 infection. Patients were divided into two groups: one control group of 21 patients receiving the national standard regimen for COVID-19, and one group of 19 patients receiving daily fingolimod (0.5 mg for 3 days) besides the standard national regimen for COVID-19 [28]. Results showed no significant differences in ventilation or mortality rates between the groups. However, fingolimod could significantly reduce the re-admission rate after hospitalization with COVID-19. In addition, the hemoglobin levels of the COVID-19 patients in the intervention group were increased compared to the controls, although the relevance of this finding remained unclear.

In summary, the articles of this topical collection have brought new insights into the roles of sphingolipids in different organ systems and have brought the field nearer to using sphingolipid-related molecules as therapeutics which will also be a main task for future research.

The contribution and effort of all authors to this collection is highly appreciated and acknowledged.
